# Central endoscopy reads in inflammatory bowel disease clinical trials: The role of the imaging core lab

**DOI:** 10.1093/gastro/gou033

**Published:** 2014-07-03

**Authors:** Harris Ahmad, Tyler M. Berzin, Hui Jing Yu, Christopher S. Huang, Daniel S. Mishkin

**Affiliations:** ^1^Medical Affairs, BioClinica Inc. Newtown, Pennsylvania, USA; ^2^Division of Gastroenterology, Beth Israel Deaconess Medical Center, Harvard Medical School, Boston MA, USA; ^3^Section of Gastroenterology, Boston Medical Center, Boston University School of Medicine, Boston, MA, USA; ^4^Division of Gastroenterology, Granite Medical Group, Boston Medical Center, Boston, MA, USA

**Keywords:** inflammatory bowel disease, clinical trials, imaging core laboratories

## Abstract

Clinical trials in inflammatory bowel disease (IBD) are evolving at a rapid pace by employing central reading for endoscopic mucosal assessment in a field that was, historically, largely based on assessments by local physicians. This transition from local to central reading carries with it numerous technical, operational, and scientific challenges, many of which can be resolved by imaging core laboratories (ICLs), a concept that has a longer history in clinical trials in a number of diseases outside the realm of gastroenterology. For IBD trials, ICLs have the dual goals of providing objective, consistent assessments of endoscopic findings using central-reading paradigms whilst providing important expertise with regard to operational issues and regulatory expectations. This review focuses on current approaches to using ICLs for central endoscopic reading in IBD trials.

## INTRODUCTION

Physicians and pharmaceutical companies designing clinical trials of experimental therapies for inflammatory bowel disease (IBD) face several challenges. Contemporary IBD trials rely on endoscopic endpoints for determining patient eligibility and drug efficacy. Mucosal healing is used as one of the most reliable objective markers for treatment efficacy [1, 2]. Historically, assessment of mucosal healing has been accomplished using static images of the small and large bowel. In recent years, there has been a predominant trend towards centralization of endoscopic evaluations relating to studies evaluating both experimental drugs and novel scoring systems in IBD. This centralization rests on assessment of full-length videos of colonoscopies and sigmoidoscopies, in order to most closely record the best view, or reflect the view of the clinical site [1, 3]. The incorporation of a video component into IBD disease evaluation creates new technical challenges for sponsors of clinical trials and research studies, since video acquisition is not yet the standard of care in clinical endoscopy suites. Compared with still images, video is more challenging to acquire and may require segmental annotation. Further, the need to archive and transmit video data from clinical sites to a central database in a standardized format creates operational challenges for all parties involved in an IBD clinical trial.

In contrast to trials in rheumatology, neuroscience and cardiology—in which objective eligibility and efficacy endpoints are typically assessed centrally by a research team—clinical trials in IBD often rely on the interpretation of endoscopic findings by local participating physicians or principal investigators (PIs), as well as on assessment by an expert gastroenterologist functioning as a central reader. This increases the chance of potential discord between official local interpretations *vs* central interpretations. While one may think that the duty endoscopist would be best equipped to provide the most accurate score, there is increasing evidence to suggest otherwise; this was highlighted in a recent review of a study on mesalamine in ulcerative colitis (UC), in which there was no statistical difference between treatment and placebo [4]. As the authors state, one possible explanation could be that 31% of the subjects enrolled by PIs should actually not have been included in the study. Although the PIs assessed these subjects as exhibiting the minimum disease activity on endoscopy required for inclusion into the study, this set of subjects were read for efficacy by the central read and found that the subjects did not have the minimum degree of disease activity on endoscopy for study eligibility. A retrospective analysis indicates that had the trial been conducted with central readers assessing subjects for eligibility and excluded these subjects, the study would have demonstrated statistically significant efficacy, with results similar to those of other trials of mesalamine. [5].

In addition to technical and operational challenges, clinical trials in IBD also lack standardized approaches to the design of centralized reading facilities for assessment of mucosal healing, compared with other fields such as oncology or rheumatology, where read design is a widely discussed and well documented topic [6–12]. These areas of clinical trials enjoy specific, documented, regulatory guidance; for example, in rheumatology there are regulatory documents guiding sponsors on the use of two independent central readers, blinded to treatment arm and time point, for assessment of structural progression of disease on serial radiographs [13]. A recent meta-analysis shows that this paradigm is widely instituted for all phases of clinical trials in rheumatology [14]. On the other hand, study designs in IBD trials vary significantly, with no consensus regarding optimal methodologies for read paradigm (image review methodology), approaches to assessment of inter- and intra-observer variability, or the appropriateness of adjudication that seeks to resolve differences in interpretation (Table 1).

**Table 1.** Local and central reading paradigms in IBD clinical trials: example study designs

Paradigm	Scoring criteria	Eligibility design		Eligibility adjudication	Efficacy design		Efficacy adjudication	Reader variability monitoring
		Local reader	Central reader		Local reader	Central reader		
1	CDEIS SES-CD [23]	Yes	1 reader	Central read stands	None	1 reader	None	None
2	CDEIS SES-CD [23]	None	None	None	None	2 readers + adjudicator	Top 10% of most discordant reads	None
3	SES-CD [4]	Yes	1 out of pool of 3 readers	If discord occurs on determination of eligibility	None	2 readers + adjudicator	Top 10% of most discordant reads	10% of total subjects re-inserted to assess intra- and inter-reader variability on eligibility and efficacy
4	Mayo Baron [22]	None	1 out of pool of 3 readers	None	None	2 readers	None	10% of total subjects re-inserted to assess intra-reader variability on efficacy
5	Mayo [25]	Yes	1 reader (if local reader scores 2 or higher)	If local reader scores 2 or higher & central reader scores 0 or 1	None	2 readers	None	10% of total subjects re-inserted to assess intra-reader variability on efficacy
6	Mayo UCEIS [3, 25]	None	1 out of pool of 3 readers; each assess 33.3%	None	None	1 out of pool of 3 readers; each assess 33.3%	None	10% of total subjects re-inserted to assess intra- and inter-reader variability on eligibility and efficacy
7	Mayo [25]	Yes	1 out of pool of 2 readers (if local reader scores 2 or higher)	If local reader scores 2 or higher & central reader scores 0 or 1	Yes; unblinded to time point	1 out of pool of 2 readers; blinded to time point	Disagreement of ≥1 between local and central reads	73 subjects (based on power analysis) re-inserted to assess intra- and inter-reader variability on eligibility and efficacy

In the pharmaceutical industry, ICLs have played a critical role in the development and approval of a wide variety of new therapies [15–21]. ICLs are likely play an increasingly important role as IBD trial methodology matures and becomes more standardized. An important first step for ICLs is to help improve the accuracy of IBD endpoints through the use of experienced, centralized readers.

For IBD trials, ICLs are taking on the important role of managing complex video data from sigmoidoscopies and colonoscopies, acquired from large multi-centered studies, in support of eligibility criteria as well as safety and efficacy endpoints.

## ENDOSCOPIC VIDEO ASSESSMENT AND ICL WORKFLOW

Assessments in IBD trials are generally based on one of several endoscopic scoring criteria, including the modified Baron’s, Mayo, UCEIS, Simple Endoscopic Scoring in Crohn’s (SES-CD), or Crohn’s Disease Endoscopic Index of Severity (CDEIS) [3, 22–26]. As an example of the ICL workflow for an IBD trial, patients enrolled with active disease undergo an endoscopic assessment at entry and at a designated follow-up after initiating therapy to assess drug efficacy. The gastroenterologist performing the initial endoscopy would record a video of the examination, which is either mailed as a hard copy to the central reading site, or directly uploaded to a central database via an FTP site. Video recording in a standardized format can be difficult, depending on the set-up of the endoscopy suite. Additionally, certain scoring systems require anatomic annotations to be embedded in the video, to ensure that central readers are scoring the same anatomic segments. Purpose-built software packages are being designed for endoscopic evaluation, which can facilitate recording, whilst allowing for annotation recording and simplified data uploads. Once a video has been analysed for resolution and quality by the ICL, it can be uploaded to a central reader’s remote workstation for review and scoring, based upon a set of pre-defined queries and parameters in the study protocol. In this scenario, video data often needs to be reviewed expeditiously (typically within 24 hours of central upload), in order to confirm decisions regarding trial eligibility.

The ICLs also have an important role in calibrating the various steps in this process and making sure that all can be completed to a minimum standard and ideally at the highest quality; this includes verifying the local site’s ability to record a high-quality video and submit or upload it to the central database. It is critical that this step be accomplished before the evaluation of a potential patient for inclusion into a trial. Study sites also rely on feedback from ICLs regarding technical factors, bowel preparation and mucosal visualization, in order to optimize video capture and data analysis. This ongoing feedback can be instrumental in standardized assessment in a global, multi-center, clinical trial.

In addition, a similar calibration is needed for the central readers. While expert readers are aware of the scoring systems in IBD, mock cases may be used prior to initiation of a trial—and potentially at specified intervals during the trial—to help ensure standardization of reader scoring and to improve inter-observer agreement. To date, experience has demonstrated that continued communication and feedback from the central readers, who are adept at evaluating endoscopic videos and findings, can better identify which sites are providing high-quality videos and opportunities for and which are revealing opportunities for improvement. 

## SPECIFIC APPROACHES TO SUPPORTING A TRIAL WITH A LOCAL AND CENTRAL READING

Historically, an experienced physician at a primary trial location will serve as Principal Investigator, providing overview of over eligibility assessment and safety monitoring. With an increased need for central reading in clinical trials by regulatory authorities, sponsors designing IBD trials face the challenge of balancing the role of a local GI endoscopist with the utilization of an experienced central reader. The balancing of the two parties has resulted in a myriad paradigms (Table 1), with some sponsors excluding local reading entirely.

For IBD trials, central readers can provide unbiased confirmation of endoscopic findings, as compared with a local PI [6], particularly in studies covering multiple sites, with limited numbers of patients enrolled at each site. The use of central readers can also yield cost savings and may contribute to more accurate assessments of therapeutic efficacy by reducing the occurrence of inappropriately enrolled subjects.

Despite the use of standardized scoring systems and appropriate training, in multi-site IBD studies there is still a risk that inter-observer variability will significantly affect data interpretation and sample size requirements. Central validation of endoscopic scores helps ensure that each patient enrolled and monitored is assessed using exactly the same criteria, irrespective of local expertise. This activity helps reduce discord between a central reader and a local physician, and creates an opportunity to implement improvements at local sites for endoscopic scoring, enrolment criteria and other key issues.

## ADVANTAGES AND DISADVANTAGES TO IBD TRIAL PARADIGMS

### Eligibility designs

The major factors influencing an eligibility design are scientific caliber, operational intricacy, and turnaround time. Sponsors and ICLs have chosen to design specific studies with custom designs based on sponsors' budgets, reducing local read bias or maximizing the rate of enrolment. For example, among the paradigms listed in Table 1, Paradigm 1 carries the advantage of employing both local and central reading for determination of eligibility, whilst avoiding adjudication by using the central read in case of a discord. This can avoid the disadvantage of a delay in determination of final eligibility, should an adjudicator be involved—which is an extra operational step in the reading process.

With turnaround time being of the utmost importance for eligibility, the advantages of Paradigms 4 and 6 carry with them the efficient approach of not utilizing local reading at all for determination of eligibility, and consigning the assessment of patient inclusion, using the Mayo scoring system, solely to the central reader. The disadvantage of the two similar paradigms is that the sponsor is only receiving one assessment of eligibility. However, if further studies confirm the findings of the previously-mentioned mesalamine study [6], in which there was over-enrolment due to local reading for eligibility, a paradigm based entirely on central reading for eligibility may in fact be superior.

Paradigms 3, 5, and 7 carry the advantage of involving the assessments of both the local and central readers, in order to achieve a higher degree of consensus between the two parties. However, involving both local and central readers demands resolution of differences of interpretation, should they occur through an adjudication read by a 2^nd^ central reader. This 2^nd^ central reader would score the subject in the same manner as the first central reader; with no knowledge of the local or 1^st^ central reader's score and with no knowledge of the subject s clinical information. The advantage of this paradigm is that it provides an opportunity to resolve a disagreement between local and central reader assessment while the disadvantage is that the paradigm involves another step in the read process and thus an additional time allowance for eligibility determination.

### Efficacy designs

Just as eligibility designs vary between single- and double assessments, so can efficacy designs. The simplest design is to read the time points using a single reader paradigm. The advantage, of course, is a cost, given that the reading of scores such as the Mayo, UCEIS, or CDEIS can be quite costly, even with just a single reading. Further, the advantage of a single read is that it avoids the need to resolve a difference of scoring that would result in a Further, the advantage of a single read is that it avoids potential differences of scoring. While Paradigms 1 and 6 utilize this strategy, they carry the disadvantage of not including a second read to confirm or refute the very important endpoint determinations of mucosal healing among IBD studies.

Paradigms 2, 3, 4, 5 and 7 all utilize a double read, with the first four designs involving two central readers and Paradigm 7 taking a similar approach as described in the eligibility scenarios, in which scoring criteria are assessed by a local and a central reader. The advantage again is the added weight of two assessments for a particular time point or points. This paradigm matches that of the numerous trials in rheumatoid arthritis, in which a double read with an adjudicator is the ‘gold standard' approach and recommended by the regulatory authorities. The disadvantage, of course, is the added expense of compensating two readers and the need to resolve any differences in scoring, either with two central readers one local and one central.

### Efficacy adjudication designs

A degree of discord is inevitable in a double read for eligibility or efficacy; before the trial begins, it is important to have a predetermined action plan to deal with it. One such approach, as utilized in Paradigms 4, 5 and 6, is to employ no adjudication at all. The advantage is, of course, reduced cost, whilst on the other hand the disadvantage is the need to resolve these sometimes severe differences in a manner acceptable for analysis. There are several such methods, such as simply averaging the scores or setting a difference in score *a priori* that results in removal of the subject from analysis. The disadvantages of these approaches are the uncertainty of whether the methodology is scientifically sound with published trial data supporting the rational.

A second approach to resolving discordant scoring is to determine a percentage of cases to be adjudicated before the start of the study. For example, Paradigms 2 and 3 employ an approach in which the top 10% of most discordant reading scores discordant reads would be assessed by a third central reader for final decision; in other words, analysis would be performed by the ICL to determine which subjects had the greatest degree of difference in change in interpretation between two central readers. If one hundred cases were included in the read, 10 cases representing the highest degree of discord on the change in severity of the subjects would be assessed by a third central reader, who would adjudicate choose which of the two central reader scores were most agreeable and this score would be the final score for the subject.

The first advantage of this paradigm is that it addresses the need to resolve scoring differences in a portion of subjects in which the scoring assessments of change in difference was the greatest. The second advantage is that it allows for a predictable method of determining the number of cases that would eventually need to be budgeted for adjudication reads. The first disadvantage is that the arbitrarily chosen percentage or number of cases may be above or below that which is scientifically necessary. The second disadvantage is that the adjudication cases cannot be assessed until the end of the study, when the first two central reads have been completed, since the percentage of subjects chosen is based on the total number of cases completed.

The first advantage of this threshold based approach is that it is specifies in advance a degree of variance, allowing for appropriate adjudication of a subject as needed. The second advantage is, of course, that adjudication can be done in a real-time fashion, instead of at the end of the trial. The disadvantage is that the budget is not capped at an expected number of adjudicated time points since theoretically a range of 0 to 100% of all subjects could meet the threshold and be allocated for this adjudication read. This could increase the sponsor budget for central reads beyond expectations while also adding a time delay for final score determination on a higher than expected reads.

## CONCLUSION

Clinical trials in IBD pose significant challenges for drug sponsors due to the incorporation of complex video data, the lack of standardized approaches to the design of central reading, as well as the difficulty of balancing local and central reads. As a result, trials in this therapeutic area use a multitude of reader paradigms, ranging from endoscopic scoring made exclusively by the local endoscopist, to scoring exclusively by the central reader, with little regulatory guidance regarding the optimal paradigm for IBD trials [27, 28]. As a response to these challenges—and armed with experience from similar therapeutic areas—ICLs are likely to play an increasingly important role in applying robust, evidence-based methodologies, while promoting more standardized approaches to endoscopic assessment by local and central readers.
